# Underlying Mechanisms of GBA1 in Parkinson’s Disease and Dementia with Lewy Bodies: Narrative Review

**DOI:** 10.3390/genes16121496

**Published:** 2025-12-15

**Authors:** Anastasia Bougea

**Affiliations:** 1st Department of Neurology, Medical School, National and Kapodistrian University of Athens, 72 Vas. Sophias Ave, 11528 Athens, Greece; abougea@med.uoa.gr; Tel.: +30-210-7289285 or +30-210-7235212; Fax: +30-210-7289474

**Keywords:** Parkinson’s disease (PD), Dementia with Lewy Bodies (DLB), glucocerebrosidase 1 (*GBA1*)

## Abstract

**Background/Objectives**: Parkinson’s disease (PD) and Dementia with Lewy Bodies (DLB) are neurodegenerative disorders characterized by the accumulation of misfolded alpha-synuclein protein in the brain. Mutations in the glucocerebrosidase 1 (*GBA1*) gene have been identified as a significant genetic risk factor for both PD and DLB. GBA1 encodes for the lysosomal enzyme glucocerebrosidase, which is responsible for the breakdown of glucosylceramide (GC). Deficiencies in glucocerebrosidase activity lead to the accumulation of glucosylceramide within lysosomes, contributing to lysosomal dysfunction and impaired protein degradation. The aim of this narrative review is to update the underlying mechanisms by which *GBA1* mutations contribute to the pathogenesis of PD and DLB. **Methods**: A comprehensive literature search was conducted across four major electronic databases (PubMed, Web of Science (Core Collection), Scopus, and Embase) from inception to 8 November 2025. The initial search identified approximately 1650 articles in total, with the number of hits from each database being as follows: PubMed (~450), Web of Science (~380), Scopus (~520), and Embase (~300). **Results**: The mechanism by which mutations in the GBA1 gene contribute to PD involves both loss-of- function and gain-of-function pathways, which are not mutually exclusive. Typically, GBA1 mutations lead to a loss of function by reducing the activity of the GCase enzyme, impairing the autophagy- lysosomal pathway and leading to α-synuclein accumulation. However, some mutant forms (*GBA1L444P*) of the GCase enzyme can also acquire a toxic gain of function, contributing to α-synuclein aggregation through mechanisms like endoplasmic reticulum stress and misfolding. While Venglustat effectively reduced GC levels, a key marker associated with GBA1-PD, the lack of clinical improvement led to the discontinuation of its development for this indication. **Conclusions**: *GBA1*-mediated lysosomal and lipid dysregulation represents a key pathogenic axis in PD and DLB. Understanding these mechanisms provides crucial insight into disease progression and highlights emerging therapeutic strategies—such as pharmacological chaperones, substrate reduction therapies, and gene-targeted approaches—aimed at restoring GCase function and lysosomal homeostasis to slow or prevent neurodegeneration.

## 1. Introduction

Parkinson’s disease (PD) and Dementia with Lewy Bodies (DLB) are neurodegenerative disorders characterized pathologically by aggregation of misfolded α-synuclein into Lewy bodies (LBs) and Lewy neurites in vulnerable regions of the brain [1]. Although both conditions share overlapping clinical and pathological features, including motor impairment, cognitive decline, and Lewy body pathology, their molecular underpinnings remain incompletely understood. A key advance in the field has been the recognition that heterozygous (and, less commonly, homozygous) mutations in the *GBA1* gene—which encodes the lysosomal enzyme glucocerebrosidase (GCase)—represent one of the most common known genetic risk factors for PD and DLB [2,3]. Large-scale genetic and neuropathological studies have since confirmed *GBA1* as the most prevalent genetic risk factor for sporadic PD and DLB, present in approximately 5–10% of PD cases and up to 20% of DLB cases [4,5], and, when affected, often show earlier onset, more rapid progression, and increased cognitive impairment compared to idiopathic cases (Table 1).

Mechanistically, *GBA1* mutations lead to reduced enzymatic activity of GCase, resulting in impaired lysosomal degradation capacity and subsequent accumulation of α-synuclein [2]. This relationship appears bidirectional: not only does reduced GCase activity promote α-synuclein aggregation, but aggregated α-synuclein itself can further inhibit GCase trafficking and function, creating a self-perpetuating pathogenic loop. Beyond this core lysosomal dysfunction, *GBA1*-related pathology exerts wide-ranging effects on cellular metabolism and homeostasis [6]. Altered sphingolipid metabolism, endoplasmic reticulum (ER) stress, mitochondrial impairment, oxidative stress, and neuroinflammation have all been implicated as downstream consequences of GCase deficiency [7]. Together, these processes contribute to synaptic dysfunction, neuronal death, and the characteristic progression of Lewy body pathology from brainstem to cortical regions [8].

Given this strong genetic association, much effort is being devoted to understanding the mechanistic links between GCase deficiency (or dysfunction) and synucleinopathy-driven neurodegeneration. The aim of this narrative review is to update and synthesize current knowledge regarding how GBA1 mutations may promote PD and DLB pathogenesis—including both “loss-of-function” and “gain-of-function” mechanisms—and to highlight therapeutic prospects and remaining gaps.

## 2. Materials and Methods

A comprehensive literature search was conducted to identify studies investigating the underlying mechanisms of *GBA1* in PD and DLB. Four major electronic databases were searched—PubMed, Web of Science (Core Collection), Scopus, and Embase—from inception to 8 November 2025.

The search combined controlled vocabulary terms (MeSH in PubMed, Emtree in Embase) and free-text keywords related to *GBA1*, glucocerebrosidase, Parkinson’s disease, dementia with Lewy bodies, and key mechanistic pathways (e.g., α-synuclein aggregation, lysosomal dysfunction, autophagy, mitochondrial dysfunction, neuroinflammation, and lipid metabolism). The detailed search strategies for each database are presented in Table 2.

## 3. Results

### 3.1. GBA1: Gene, Enzyme Structure, Physiology

The *GBA1* gene is located on chromosome 1q21 and spans 11 exons and 10 introns (~7.6 kb in length). It has a highly homologous pseudogene (GBAP) approximately 16 kb downstream, which predisposes to recombination events [9]. The gene encodes the lysosomal enzyme glucocerebrosidase (GCase; ~62 kDa), which hydrolyses the glycosphingolipid substrate glucosylceramide (GlcCer) into ceramide and glucose, and also processes glucosylsphingosine (GlcSph) to sphingosine and glucose [9]. Structurally, GCase comprises three functional domains and uses catalytic residues (e.g., Glu235, Glu340, Cys342) with multiple N-linked glycosylation sites (Asn19, Asn59, Asn146, Asn270, Asn462) which affect folding, stability and lysosomal targeting [9]. In physiological conditions, the newly synthesized enzyme in the endoplasmic reticulum (ER) binds to the chaperone-like receptor LIMP2 (lysosomal integral membrane protein 2), traffics through the Golgi, and is delivered to lysosomes, where the acidic pH allows dissociation from LIMP2 and activation of the enzyme in the presence of its co-factor saposin C and appropriate lipid microenvironment [10].

Deficiency of GCase activity (in the context of biallelic *GBA1* mutations) causes the autosomal-recessive lysosomal storage disorder Gaucher disease (GD), typified by hepatosplenomegaly, bone involvement, cytopenias, and in the neuronopathic forms (types 2 and 3), severe neurodegeneration [11]. Importantly, even heterozygous carriers of GBA1 mutations—who typically do not manifest GD—have elevated risk for PD and DLB, which indicates that partial GCase deficiency (or dysfunction) in the brain is sufficient to modify risk of synucleinopathy [11].

### 3.2. GBA1 Mutations and Pd/Dlb: Epidemiology and Phenotype

Numerous *GBA1* variants (over 400–500 different mutations) have been reported, including missense changes, small insertions/deletions, splice-site and recombinant alleles. Among the more frequent PD-associated *GBA1* variants are *N370S*, *L444P*, *D409H* [12]. Residual GCase activities vary across models and assays. *N370S* is typically mild with partial enzyme function (~7–38%), while *L444P* is more severe (~13–24%). Risk alleles like *E326K* and *T369M* often show near-wildtype or mildly reduced activity [13]. Recombinant or truncating variants (e.g., *RecNciI*, *84insGG*) often show negligible enzyme activity (Table 3).

Heterozygous *GBA1* mutation carriage is estimated in ~5–10% of unselected PD cases in the general population, and up to ~15–30% in selected cohorts (e.g., Ashkenazi Jewish) [10]. Clinically, PD patients with *GBA1* mutations (GBA-PD) tend to have: earlier age at onset (typically 5–10 years earlier), more frequent cognitive impairment and dementia, more rapid progression of both motor and non-motor features, increased burden of Lewy pathology, and in some cases increased autonomic dysfunction [15,16].

Similarly, *GBA1* mutation carriers are at elevated risk of DLB, which shares overlapping pathology with PD (i.e., Lewy body disease) and is characterized by early cognitive impairment, visual hallucinations, fluctuations and parkinsonism. The precise penetrance of *GBA1* variants is variable and influenced by modifier genes, environmental exposures and other risk factors; not all carriers develop PD or DLB, indicating that *GBA1* is a risk factor rather than a deterministic gene [17]. These epidemiological and clinical observations raise important mechanistic questions: How does GCase deficiency or dysfunction contribute to α-synuclein aggregation, neuronal vulnerability, and brain–LB pathology? Below, we explore the major mechanistic domains.

### 3.3. Mechanism 1: Loss-of-Function of Gcase → Lysosomal/Autophagic Impairment

A central and widely supported hypothesis posits that *GBA1* mutations reduce GCase enzymatic activity and thereby compromise lysosomal glycosphingolipid catabolism. The downstream effect is dysfunction of the autophagy-lysosomal pathway (ALP), impaired clearance of proteins and lipids, and ultimately accumulation of α-synuclein aggregates.

#### 3.3.1. Evidence for Gcase Deficiency

Several studies show that GCase enzyme activity is reduced in carriers of *GBA1* mutations with PD compared to controls, and even reduced in some idiopathic PD (iPD) cases without known *GBA1* mutations, suggesting GCase might play a broader role [18]. For example, one review reports that GCase activity is reduced to ~58 % of normal in GBA1-PD, ~67 % in idiopathic PD, and <15 % in GD patients [10]. This finding suggests that GCase and its related pathways play a broader role in the pathogenesis of PD beyond a specific genetic link, possibly through the accumulation of alpha-synuclein, mitochondrial dysfunction, or other lysosomal issues [19].

Partial inhibition of the GCase in a mouse model of a synucleinopathy (PrP-*A53T*-SNCA) led to an increase in soluble alpha-synuclein and worsened behavioural problems [20,21]. This indicates that impaired GCase function contributes to the accumulation of toxic α-synuclein and neurodegeneration, a finding that is consistent with the role of GCase in lysosomal function and PD risk. Conversely, overexpression or viral delivery of GCase ameliorated α-synuclein accumulation and preserved dopaminergic markers. This is because a functional GCase is essential for clearing alpha-synuclein aggregates, and a deficiency in GCase leads to a buildup of these proteins, causing damage to dopaminergic neurons, as seen in diseases like Parkinson’s [22,23].

#### 3.3.2. Lysosomal/Autophagy Dysfunction

Reduced GCase activity leads to accumulation of its substrates (e.g., GlcCer, GlcSph) in lysosomes, altering lipid composition of membranes, disrupting trafficking, acidification, and hydrolase activity [10]. Disruption of autophagy (macroautophagy, chaperone-mediated autophagy—CMA) has been observed in models of GCase deficiency. For example, decreased lysosomal proteolysis (~40% reduction) was seen in GCase knockdown models [24]. The impaired ALP results in less efficient clearance of α-synuclein and other aggregation-prone proteins.

Moreover, GCase deficiency may impair lysosomal membrane stability and lead to leakage of hydrolases, triggering downstream toxicity [25]. Lipid accumulation can also affect vesicular trafficking (endosomal–lysosomal pathway), contributing to neuronal dysfunction. Notably, there appears to be a bidirectional “vicious cycle” between GCase deficiency and α-synuclein aggregation: GCase deficiency → impaired α-synuclein clearance → α-synuclein accumulation → further inhibition of GCase activity (for example by binding of α-synuclein to GCase or interfering with its trafficking) → further substrate accumulation, lysosomal dysfunction and α-syn propagation. Thus, the loss-of-function model posits that reduced GCase activity is a key upstream factor that sets the stage for synucleinopathy via lysosomal failure [26].

### 3.4. Mechanism 2: Lipid Metabolism, Substrate Accumulation and A-Synuclein Aggregation

In parallel to the ALP impairment, an important axis of the *GBA1*–PD/DLB link concerns altered lipid homeostasis, especially of glycosphingolipids like glucosylceramide (GlcCer) and glucosylsphingosine (GlcSph) [27]. When GCase is deficient, its substrates GlcCer and GlcSph accumulate inside lysosomes and other compartments. Studies report elevated levels of these lipids in brain tissue, cell models and animal models of GCase deficiency [19]. These lipid changes may alter the biophysical properties of membranes—for example, increasing membrane curvature or creating lipid microdomains that predispose to protein aggregation. In particular, GlcCer has been shown to facilitate the formation of proteinase K–resistant α-synuclein and accelerate its aggregation [28]. A mechanistic link emerges: the accumulation of glycosphingolipids such as GlcCer and GlcSph may directly promote α-synuclein fibrillization by stabilizing oligomeric or misfolded α-syn species, perhaps by providing a lipid interface or scaffold that nucleates aggregation. For example, lipid extracts from *GBA1 L444P* fibroblasts accelerated α-synuclein aggregation compared to controls in vitro [29]. Moreover, these lipid alterations may alter vesicle dynamics and membrane trafficking, impairing synaptic vesicle homeostasis (a normal function of α-synuclein) and thereby promoting synaptic dysfunction and neuronal stress.

Accumulated lipids can change the composition and fluidity of endosomal/lysosomal membranes, affecting the function of membrane-bound receptors, vesicular fusion, and trafficking of α-synuclein for degradation. In murine models, a decrease in *GCase* activity due to genetic mutations disrupts lysosomal membrane composition and integrity, leading to impaired lysosome function and altered endocytic trafficking [30]. Specifically, GCase deficiency results in the accumulation of certain lipids like GlcCer, changes in lysosomal pH, and a reduced ability to form new, functional lysosomes through a process called autophagic lysosome reformation (ALR). This disruption results in the accumulation of lipids and other substrates within lysosomes, affecting cellular processes like autophagy and contributing to the pathology of neurodegenerative diseases [14]. Thus, substrate accumulation and lipid dysregulation provide a plausible mechanistic bridge between GCase deficiency and the enhanced propensity for α-synuclein to misfold, aggregate and seed pathology.

### 3.5. Mechanism 3: Gain-of-Function Effects—Misfolded Gcase, Er Stress, Trafficking Defects

Beyond the loss-of-function model, there is growing recognition that certain mutant forms of GCase may exert “gain-of-function” toxic effects, particularly via misfolding, ER retention, triggering unfolded protein response (UPR), ER-Golgi trafficking impairment, and quality control overload [31].

Many *GBA1* mutations (e.g., *L444P*) result in misfolded GCase protein that fails to properly traffic through the ER and Golgi to lysosomes. These misfolded GCase molecules can accumulate in the ER, bind chaperones, and activate ER stress pathways and UPR [32]. ER stress can in turn impair normal cellular homeostasis, lead to activation of pro-apoptotic signalling, and perturb vesicular trafficking including ER-Golgi transport of other proteins (including α-synuclein). α-Synuclein accumulation in the ER can further impede ER–Golgi transport, which further hinders ER-Golgi transport and can contribute to cell death [33].

Misfolded GCase may reduce the amount of functional enzyme reaching lysosomes, compounding the loss-of-function effect. In addition, the burden on ER quality control and the resulting stress may impair the trafficking of other lysosomal proteins or chaperone-mediated autophagy pathways. The combined effects of the loss of GCase activity and the widespread stress caused by misfolded proteins contribute to a more significant cellular problem with protein balance, known as proteostasis impairment [14].

Recent work indicates that mutant GCase may influence not just lysosomal and ER function, but also mitochondrial function (see below). GCase has been detected in mitochondrial compartments and its deficiency may impair mitochondrial complex I, reactive oxygen species (ROS) generation, and mitochondrial morphology [34]. Thus, a gain-of-function (toxic misfolded protein) mechanism complements the loss-of-function model, and both may act in concert. Indeed, evidence propose that in GBA1-PD both mechanisms are simultaneously operative: reduced GCase activity and accumulation of misfolded GCase in the ER/trafficking compartments, which then further compromises the cellular and lysosomal homeostasis [35].

### 3.6. Mechanism 4: A-Synuclein Homeostasis, Aggregation, Seeding and Propagation

A key downstream target of the *GBA1*-linked pathogenic cascade is α-synuclein: its homeostasis, clearance, aggregation into toxic oligomers and fibrils, and propagation across neural networks. Because GCase deficiency impairs lysosomal/ACMP/CMA pathways, α-synuclein—which is degraded via autophagy/lysosomal systems—accumulates. For instance, GCase knockdown reduced proteolysis by ~40% and increased α-synuclein (by about 1.8-fold) [36]. This accumulation often involves the stabilization of toxic, soluble oligomeric intermediates, which eventually form insoluble aggregates characteristic of Lewy bodies.

Elevated levels of α-synuclein, in turn, can further inhibit the activity and trafficking of normal GCase to the lysosome, creating a “vicious cycle” that exacerbates the pathology. In Murine in vivo experiments, partial inhibition of GCase increased soluble α-synuclein and worsened motor/cognitive phenotypes; conversely, enhancing GCase activity reduced α-syn pathology [37].

As noted above, accumulating GlcCer/GlcSph can promote α-synuclein misfolding by providing a conducive environment (lipid interface, altered membrane microdomains) for assembly of α-synuclein fibrils [38]. Emerging evidence suggests that α-synuclein oligomers/fibrils can seed new aggregation in neighbouring cells (prion-like spread). In models of *GBA1*-PD (e.g., iPSC derived midbrain organoids), retention of mutant GCase in the ER and increased GlcCer were determinants of α-synuclein fibrillary aggregates with seeding activity [39]. In *GBA1* mutation carriers, impaired GCase function may accelerate not only initial accumulation of α–synuclein but also its cell-to-cell propagation, thereby driving earlier onset and more rapid progression of Lewy body pathology.

The interplay between impaired GCase function, substrate accumulation, α-synuclein aggregation and further inhibition of GCase (by α-synuclein binding or mislocalized GCase) may generate a positive feedback loop that accelerates pathology [40]. Some authors call this the “lysosomal failure–α-synuclein propagation vicious cycle” [41]. In other words, *GBA1* mutations may shift the kinetic equilibrium of α-syn clearance/accumulation towards earlier and more accelerated aggregation and spread.

### 3.7. Mechanism 5: Mitochondrial Dysfunction, Oxidative Stress and Other Organellar Cross-Talk

Beyond lysosomes and ER, GCase deficiency and *GBA1* mutation carry consequences for mitochondria and other organelles, contributing to neuronal vulnerability. Recent evidence indicates that reduced GCase activity affects mitochondrial complex I activity, increases reactive oxygen species (ROS) generation, and leads to morphological abnormalities of mitochondria. A recent review noted that *GBA1* mutations can impact (i) lysosomes and metabolites, (ii) mitochondrial complex I, and (iii) other organelles such as ER, with the first two reflecting loss-of-function and the third gain-of-function effects [10].

In a mouse GBA1 *L444P* knock-in model, mitochondrial defects were observed in dopaminergic neurons and increased susceptibility to the MPTP neurotoxin. The mutation leads to reduced GBA enzyme activity and protein levels, and an accumulation of α-synuclein, which primes the neurons to be more vulnerable to the toxic effects of MPTP, particularly mitochondrial dysfunction and cell death [42].

Lysosomal dysfunction can impair mitophagy (selective autophagic removal of damaged mitochondria) and thus promote accumulation of dysfunctional mitochondria. Impaired mitophagy is a recognized mechanism in PD pathogenesis. Thus, by compromising autophagy, GCase deficiency indirectly impacts mitochondrial quality control [43]. Endosomal trafficking defects may impair delivery of nutrients and receptor recycling, contributing to overall cell stress and degeneration in synucleinopathy contexts. Moreover, lipid accumulation (e.g., GlcCer) may alter mitochondrial membranes or signalling, further impairing mitochondrial dynamics (fusion/fission) or bioenergetics. The altered membrane properties can interfere with essential processes like oxidative phosphorylation and the regulation of mitochondrial quality control [44]. The combination of α-syn aggregation, impaired quality control (lysosomal, mitochondrial), and oxidative stress likely underlies the heightened neuronal vulnerability in *GBA1*-PD. ER stress (from misfolded GCase) may affect mitochondrial-ER contact sites and calcium homeostasis, leading to further mitochondrial insult [45]. Endosomal trafficking defects may impair delivery of nutrients and receptor recycling, contributing to overall cell stress and degeneration in synucleinopathy contexts.

### 3.8. Mechanism 6: Neuroinflammation, Glial and Immune Responses

An emerging layer in *GBA1*-linked pathogenesis concerns neuroinflammation and glial dysregulation. GCase deficiency may increase the sensitivity of glia and microglia to insults and promote pro-inflammatory states, which in turn may amplify α-synuclein-mediated toxicity. In murine models, decreased GCase activity (e.g., by CBE exposure) resulted in up-regulation of complement component C1q and glial activation, consistent with microglial involvement in the degenerative process [20]. *GBA1*-PD patients show elevated immune markers in plasma and brain tissue, and associations between certain HLA alleles and PD risk further implicate immune/inflammatory contributions [46]. GCase deficiency in glial cells may impair lysosomal lipid clearance and autophagy in glia, leading to accumulation of substrates, release of inflammatory mediators, and reduced support for neurons. The altered lipid microenvironment may influence glial-neuron interactions, synaptic pruning and neuroinflammation [47]. Because α-synuclein aggregation and spread can engage immune responses (e.g., microglial phagocytosis of α-syn monomers or aggregates), the combination of GCase-deficient glia and heightened α-syn pathology may lead to a feed-forward loop: more aggregates → more inflammation → more neuronal damage → more aggregate release. Consequently, in the *GBA1*-PD context, neuroinflammation is not simply a downstream epiphenomenon but may contribute actively to disease progression and severity.

### 3.9. Therapeutic Implications: Targeting Gcase and Downstream Pathways

Understanding the mechanistic links between *GBA1* and synucleinopathy has driven development of therapeutic strategies centred on GCase augmentation, substrate reduction, chaperone therapy, gene therapy, and modification of downstream pathways (autophagy, lysosomes, lipids).

#### 3.9.1. Gcase Augmentation in Animal and Human Cells Models

Given that GCase activity is reduced in *GBA1*-PD (and even in some iPD), interventions aimed at increasing GCase activity (either by enhancing wild-type GCase or delivering functional enzyme) are appealing. Preclinical data support that in *GBA1* mutant mouse models or synucleinopathy models, viral delivery of *GBA1* increased GCase activity, decreased GlcCer/GlcSph, reduced α-syn aggregation and ameliorated behavioural/neurodegenerative changes. Table 4 summarizes the main findings of GCase activity in PD related animal and human cells models.

However, translating this approach to the human CNS is challenging (blood–brain barrier delivery, cell-type targeting, long-term safety).

#### 3.9.2. Substrate Reduction Therapy (Srt)

Reducing synthesis of GlcCer (e.g., via glucosylceramide synthase inhibitors) is also under investigation. Venglustat (a GCS inhibitor) effectively reduced GC levels in *GBA1*-PD but did not yield clinical improvement in symptomatic patients, leading to discontinuation for that indication. A phase 1 trial [57] evaluated Venglustat in healthy volunteers, and a recent phase 2 trial (NCT02906020, MOVES-PD) tested it in patients with PD who were heterozygous for *GBA* mutations. Although Venglustat proved successful in lowering GlcCer levels in cerebrospinal fluid (CSF), which indicates target engagement, it found no improvement in Society–Unified Parkinson’s Disease Rating Scale [MDS-UPDRS]) [58]. This disconnect highlights several important mechanistic considerations. First, while substrate accumulation is a key biochemical feature of GBA1-related pathology, misfolded GCase, impaired lysosomal trafficking, mitochondrial dysfunction, and neuroinflammatory responses may independently contribute to neurodegeneration and would not be corrected by substrate reduction alone. Second, reductions measured in CSF may not reflect sufficient modulation of intracellular and membrane-associated lipid microdomains within the most vulnerable neuronal populations, where GlcCer and related sphingolipids promote α-synuclein misfolding and aggregation. Additionally, by the time symptomatic individuals are enrolled in trials, α-synuclein propagation, synaptic loss, and circuit dysfunction may already be self-sustaining and therefore less responsive to metabolic pathway intervention. Finally, variable penetrance of GBA1 mutations suggests that additional genetic or environmental modifiers drive disease expression, and thus monotherapy targeting substrate synthesis may be insufficient. Together, these findings indicate that although BBB penetration is necessary, effective disease modification will likely require earlier treatment windows and multimodal approaches that address the full spectrum of pathogenic mechanisms.

#### 3.9.3. Pharmacological Chaperones

Certain small-molecule chaperones, such as ambroxol, have shown promising potential in stabilizing misfolded glucocerebrosidase (GCase), a lysosomal enzyme whose dysfunction is implicated in PD and GBA1-associated PD [56]. These pharmacological chaperones bind to the misfolded enzyme, facilitating its proper folding and trafficking from the endoplasmic reticulum to the lysosome, where it can perform its normal catalytic function [59]. By restoring lysosomal GCase activity, these compounds may help reduce the accumulation of toxic substrates and alleviate downstream cellular stress. Preclinical studies using induced pluripotent stem cell (iPSC)–derived neurons and other cellular models have demonstrated that chaperone treatment can rescue GCase function and lead to a significant reduction in α-synuclein aggregation, a key pathological hallmark of PD [60]. Clinical trials are underway to investigate ambroxol’s effects on PD, and early trials have shown that ambroxol can increase GCase protein levels and affect α-synuclein levels in the cerebrospinal fluid [56].

#### 3.9.4. Gene Therapy for *GBA1* Mutations

Adeno-associated virus (AAV)-mediated delivery of *GBA1* (or other vectors) to the CNS is being explored. This gene therapy approach seeks to introduce a functional copy of the *GBA1* gene directly into neurons and glial cells, enabling sustained expression of active GCase. By restoring or augmenting GCase activity, the therapy is expected to reduce the accumulation of glucosylceramide and other lysosomal substrates that contribute to cellular stress and impaired proteostasis. In addition, enhanced lysosomal function may facilitate the clearance of misfolded α-synuclein, a toxic protein closely linked to PD pathogenesis, thereby slowing or even halting progressive neuronal loss [61]. Preclinical studies in rodent and non-human primate models have shown encouraging results, including improved GCase activity, reduced α-synuclein pathology, and neuroprotection [51]. However, translating these findings to humans remains at an early stage. Challenges include achieving widespread and safe CNS delivery, ensuring long-term expression without immune responses, and determining optimal dosing strategies. Ongoing early-phase clinical trials and vector optimization efforts will be crucial in establishing whether AAV-based *GBA1* gene therapy can provide a durable and disease-modifying treatment for PD and related synucleinopathies.

#### 3.9.5. Enhancing Autophagy/Lysosome Function and Mitochondrial Support

Given the broader dysfunction in autophagy/lysosomal and mitochondrial pathways, drugs that enhance autophagy (e.g., mTOR inhibitors, AMPK activators), boost mitochondrial biogenesis or reduce oxidative stress may complement GCase-centred therapies. Moreover, targeting neuroinflammation (e.g., microglial modulators) may help break the self-amplifying cycle of α-syn aggregation, substrate accumulation and glial activation.

#### 3.9.6. Biomarker Development

Given the mechanistic insights, biomarkers are under exploration: GCase activity in CSF or blood, GlcCer/GlcSph levels, α-synuclein oligomers, lysosomal protein markers, neuroinflammatory signatures and imaging of lysosomal/mitochondrial dysfunction [10]. Reliable biomarkers of GCase deficiency are critical for patient stratification and assessing target engagement in *GBA1*-associated PD. CSF GCase activity and lysosomal substrates (GlcCer/GlcSph) provide the most direct measures of CNS enzyme function, while CSF α-synuclein seed amplification assays (SAA) reflect ongoing pathogenic aggregation [62,63]. Peripheral measures, including plasma sphingolipids or leukocyte GCase activity, are useful for pharmacodynamics but imperfect surrogates for brain pathology [64]. Neurofilament light (NfL) and imaging markers (DAT-SPECT, MRI) can track neurodegeneration and serve as surrogate endpoints for progression [65]. These biomarkers may help stratify *GBA1* carriers, monitor therapy response and identify at-risk individuals for early intervention. However, more longitudinal studies are needed to confirm these results.

## 4. Discussion

The pathogenesis of *GBA1* mutations in PD and DLB involves multiple converging and interlocking mechanisms. The GBA1 gene encodes the enzyme GCase, and its dysfunction leads to PD and DLB through pathways that include both loss-of-function and toxic gain-of-function effects. GBA1 mutations typically cause a reduction in the enzymatic activity of GCase. Autophagy-Lysosomal Pathway (ALP) impairment compromises lysosomal capacity, leading to dysfunction of the ALP (macroautophagy, chaperone-mediated autophagy) and resulting in the impaired clearance of α-synuclein and its accumulation. A bidirectional pathogenic loop exists where GCase deficiency leads to α-synuclein accumulation, and the accumulated α-synuclein, in turn, further inhibits GCase activity and trafficking, accelerating pathology. GCase deficiency causes the accumulation of its substrates, primarily GlcCer and GlcSph, within the lysosomes and other cellular compartments. The elevated levels of these glycosphingolipids can directly promote α-synuclein pathology by providing a lipid interface that facilitates the misfolding, aggregation, and fibrillization of α-synuclein. According to the Gain-of-Function and Cellular Stress, certain severe mutations (e.g., *L444P*) cause the GCase protein to misfold. These misfolded proteins are retained in the ER, activating the ER stress pathways and the Unfolded Protein Response (UPR), leading to pro-apoptotic signalling and general proteostasis impairment.

GCase deficiency impairs mitochondrial complex I activity and increases Reactive Oxygen Species (ROS) generation. Lysosomal dysfunction also compromises mitophagy (the clearance of damaged mitochondria), further contributing to neuronal vulnerability. GCase deficiency in glial cells (microglia/astroglia) can impair their function and promote a pro-inflammatory state that amplifies α-synuclein-mediated toxicity. Impaired GCase function accelerates the cell-to-cell propagation (or “prion-like spread”) of α-synuclein pathology, which drives earlier onset and more rapid disease progression. These mechanisms align with the clinical features of *GBA1*-associated PD/DLB: earlier onset, accelerated progression, and more prominent cognitive involvement.

Therapeutically, these insights have spurred development of GCase-centred strategies (enzyme augmentation, chaperones, substrate reduction, gene therapy) as well as approaches targeting autophagy, mitochondria and inflammation. However, translation to effective disease-modification in humans remains challenging—recent negative trial results (such as with Venglustat) underscore the complexity of the biology and the necessity of informed biomarker-driven trial designs, early intervention windows, and combinatory therapeutic strategies. This raises key questions:

- Is substrate reduction alone sufficient, or is the timing too late (i.e., after neurodegeneration is advanced)?
- Are there cell-type differences (neurons vs. glia) or region-specific vulnerabilities that require targeted delivery?
- Is GCase deficiency necessary and sufficient, or only a modifier in a multi-step pathogenic cascade?
- How to cross the blood–brain barrier effectively, target the correct brain regions, and achieve sustained effects?
- Given the heterogeneity of GBA1 variants (and differences in residual GCase activity), personalized approaches may be needed.

### Future Directions and Challenges

While considerable progress has been made in delineating GBA1-linked mechanisms in synucleinopathy, many gaps remain. Not all GBA1 variants are equal: some (e.g., *L444P*) or complex alleles are typically associated with markedly reduced GCase activity, leading to earlier disease onset, faster progression, and a higher prevalence of cognitive impairment or DLB [66]. In contrast, milder variants like *N370S* tend to confer a lower risk and later onset of PD, often with a more slowly progressive phenotype. Homozygous or compound heterozygous individuals generally display more severe clinical outcomes than heterozygous carriers, consistent with a gene dosage effect, where cumulative loss of GCase function exacerbates lysosomal dysfunction and α-synuclein accumulation. However, emerging evidence suggests that the relationship between residual GCase activity and PD risk is not linear [67]. Some individuals with comparable enzyme activity show differing disease susceptibility, implying that additional genetic, environmental, or cellular factors modulate disease expression. Variants affecting GCase stability, trafficking, or interaction with α-synuclein may exert distinct pathogenic effects beyond enzyme kinetics. Understanding these variant-specific mechanisms is crucial for developing tailored therapeutic approaches and predicting individual responses to GCase-targeted therapies such as chaperones or gene therapy.

Cell-type vulnerability in *GBA1*-associated synucleinopathies is attributed to intrinsic metabolic stress and network dysfunction, not just GCase deficiency. Dopaminergic neurons, due to their metabolic needs and reliance on calcium signalling, are particularly susceptible to oxidative stress, leading to α-synuclein misfolding with declining GCase activity. However, evidence shows that vulnerability includes limbic and cortical neurons impacted by disrupted sphingolipid metabolism, resulting in α-synuclein aggregation and synaptic issues. Glial dysfunction exacerbates this vulnerability, with reactive astrocytes and impaired microglia contributing to neuroinflammation and aggregate persistence. Notably, loss of *GBA1* in oligodendrocytes negatively affects myelination and promotes α-synuclein inclusion, indicating that glial GCase deficiency impacts network stability. Overall, *GBA1*-linked pathology arises from unique cell-type vulnerabilities to stressors, necessitating targeted therapeutic approaches [27].

Deficiency of GCase establishes a bidirectional pathogenic link with α-synuclein pathology in PD and DLB. Reduced GCase activity results in glucosylceramide accumulation, destabilizing lipid homeostasis and promoting α-synuclein misfolding, which in turn interferes with GCase function, creating a feedback loop of dysfunction. These biochemical changes may precede clinical symptoms in *GBA1* mutation carriers, highlighting a therapeutic window for restoring lysosomal homeostasis to prevent neurodegeneration. Enhancing GCase activity has shown disease-modifying potential, particularly if initiated early, though current clinical efficacy is affected by intervention timing and genetic variability. Diminished GCase activity also appears in iPD, indicating that therapies targeting GCase function and lysosomal metabolism could benefit both genetic and sporadic PD cases. A multimodal approach combining GCase augmentation with autophagy enhancers, mitochondrial stabilizers, and anti-inflammatory strategies may be required to address the interlocking pathologies of lysosomal failure, mitochondrial dysfunction, and neuroinflammation. Developing standardized, validated biomarker panels to monitor these processes remains a key priority for translation into precision therapeutics.

Research on *GBA1* primarily focuses on PD, but its role in DLB is less understood [68,69]. Recent evidence questions the assumption that *GBA1*-related lysosomal dysfunction causes a uniform synucleinopathy, revealing distinct regional effects and disease paths. In *GBA1* carriers, GCase deficiency leads to α-synuclein misfolding, particularly affecting cortical neurons, which may be more susceptible to metabolic disruptions that worsen disease expression [70]. Neuropathologically, *GBA1*-linked DLB shows greater cortical Lewy body accumulation and more rapid cognitive decline compared to sporadic DLB [70] and short survival [71]. This suggests that GCase deficits increase vulnerability in non-dopaminergic networks. Furthermore, glial dysfunction, including reactive astrocytosis and inadequate microglial cleanup, exacerbates cortical issues. The differences between PD and DLB in *GBA1* carriers indicate that factors like bioenergetic demands and immune responses are critical in determining the disease phenotype. Thus, there is a pressing need for research into the specific consequences of GCase loss and developing therapies to restore lipid balance and lysosomal function in vulnerable brain regions. However, there are several challenges in measuring GCase activity, a lack of effective model systems, the complexity of how GCase dysfunction contributes to DLB, and the blood–brain barrier (BBB) limiting therapy delivery. Specifically, the presence of other enzymes makes measuring GCase activity in vitro difficult, while creating models that fully replicate DLB pathology remains a hurdle [31]. Furthermore, GCase mutations can have poor penetrance, and therapies must overcome the BBB to be effective.

## 5. Conclusions

The *GBA1*–GCase axis provides a compelling mechanistic link between lysosomal lipid metabolism and α-synucleinopathies and holds promise for stratified therapeutic approaches. Further work is needed to refine our understanding of variant-specific effects, temporal dynamics, regional vulnerability, and the effective translation of mechanistic knowledge into clinical benefit.

## Figures and Tables

**Table 1:** Summary of overlapping clinical and pathological features of PD and LB.

Clinical/Pathological Features	PD	DLB
Motor symptoms	Bradykinesia, rigidity, tremor	Similar, but often milder tremor
Cognitive decline	Late- stage feature	Early and prominent
Hallucinations	Less frequent early	Common and early
Lewy bodies	Brainstem predominant	Cortical and limbic predominant
*GBA1* association	strong	strong

PD: Parkinson’s disease; DLB: Dementia with Lewy Bodies.

**Table 2:** Search strategies for literature review.

Database	Search Syntax	Number of Hits	Notes
**PubMed**	((“GBA1” [All Fields] OR “glucocerebrosidase” [MeSH Terms] OR “glucocerebrosidase” [All Fields] OR “GBA mutation*” [All Fields]) AND (“Parkinson Disease” [MeSHTerms] OR “Parkinson’s disease” [All Fields] OR “Dementia with Lewy Bodies” [MeSH Terms] OR “Lewy body dementia” [All Fields]) AND (“pathophysiology” [Subheading] OR “mechanism*” [All Fields] OR “lysosomal dysfunction” [All Fields] OR “alpha-synuclein” [All Fields] OR “neurodegeneration” [All Fields] OR “autophagy” [All Fields] OR “mitochondrial dysfunction” [All Fields]))	~450	Combines MeSH + free text; covers both PD & DLB.
**Web of Science (Core Collection)**	TS = ((“GBA1” OR “glucocerebrosidase” OR “GBA mutation*” OR “GBA variant*”) AND (“Parkinson’s disease” OR “Parkinson disease” OR “Dementia with Lewy bodies” OR “Lewy body dementia” OR “Lewy body disorder*”) AND (“mechanism*” OR “pathophysiology” OR “lysosomal dysfunction” OR “alpha-synuclein” OR “synuclein aggregation” OR “autophagy” OR “mitochondrial dysfunction” OR “lipid metabolism” OR “neuroinflammation”))	~380	Topic search (title/abstract/keywords) in WoS; good for citation tracking.
**Scopus**	TITLE-ABS-KEY ((“GBA1” OR “glucocerebrosidase” OR “GBA mutation*” OR “GBA gene” OR “GBA variant*”) AND (“Parkinson’s disease” OR “Parkinson disease” OR “Dementia with Lewy bodies” OR “Lewy body dementia” OR “Lewy body disorder*”) AND (“mechanism*” OR “pathophysiology” OR “alpha-synuclein” OR “synuclein aggregation” OR “lysosomal dysfunction” OR “autophagy” OR “mitochondrial dysfunction” OR “neuroinflammation” OR “lipid metabolism” OR “protein misfolding”))	~520	Broad coverage; includes conference papers; may retrieve extra non-mechanistic studies.
**Embase**	(‘gba1’/exp OR ‘glucocerebrosidase’/exp OR ‘gba mutation’:ab,ti OR ‘gba variant’:ab,ti) AND (‘parkinson disease’/exp OR ‘parkinson’s disease’:ab,ti OR ‘dementia with lewy bodies’/exp OR ‘lewy body dementia’:ab,ti) AND (‘pathophysiology’/exp OR ‘mechanism*’:ab,ti OR ‘alpha synuclein’/exp OR ‘synuclein aggregation’:ab,ti OR ‘lysosomal dysfunction’:ab,ti OR ‘autophagy’/exp OR ‘mitochondrial dysfunction’:ab,ti OR ‘lipid metabolism’:ab,ti OR ‘neuroinflammation’/exp)	~300	Using Emtree terms plus abstracts/titles; useful for pharmacology/therapeutic mechanism side as well.

Searches were limited to English-language articles involving human, animal, or cellular models relevant to disease mechanisms. Reference lists of key articles and reviews were hand-searched to identify additional publications.

**Table 3:** *GBA1* Variants and Reported Residual GCase Activities.

Variant (Protein)	Classification	Reported Residual GCase Activity (%)	Role of the Protein Variant
***N370S* (N409S)**	Mild GD/PD risk	~7–38% (reports vary by model/assay; cell lines: ~7.7% in Navarro-Romero [14]; clinical/biochemical ranges reported 32–38%).	A common *GBA1* variant often associated with a milder form of GD or increased risk for PD. Leads to reduced, but often still substantial, GCase activity.
***L444P* (L483P)**	Severe GD/higher PD risk	~13–24% (classic GD severe range)—cell lines: ~15.9% reported; ranges vary by assay.	A severe *GBA1* variant commonly associated with the neuronopathic forms of GD and a higher risk for PD. Significantly impairs GCase folding and/or trafficking.
***E326K* (E365K)**	Risk/modifier variant (non-GD in many cases)	Mild reduction or near-wildtype in some assays; considered a risk/modifier allele rather than classic GD-causing.	Generally considered a risk factor for PD or a modifier of GD severity, rather than a primary GD-causing mutation. Has a minimal effect on GCase activity.
***T369M* (T408M)**	Risk/modifier variant	Variable; often reported as mild effect on activity or stability.	Similar to *E326K*, often acts as a risk factor for PD or a modifier allele. Causes a mild, variable reduction in GCase function or stability.
***D409H* (D448H)**	Severe/GD-associated	Reported to markedly reduce activity (large reductions; often in severe GD category).	A severe *GBA1* variant often associated with a severe phenotype of GD. Causes a marked reduction in GCase activity, likely through destabilization or impaired function.
***R120W***	Reported in PD/GD cohorts	Variable; often results in reduced activity but wide ranges reported.	Identified in both GD and PD patients. Leads to reduced GCase activity, likely due to impaired enzyme stability or function, with variable clinical severity.
***V394L***	Reported in PD/GD cohorts	Reported reductions; magnitude varies by study.	Identified in both GD and PD cohorts. Causes a reduction in GCase activity, the extent of which can vary, influencing phenotype.
***RecNciI*** **/Recombinant alleles (e.g., RecTL, *RecNciI*)**	Complex recombinant alleles (often severe)	Often associated with severely reduced activity and severe GD phenotype; associated with increased PD risk.	Complex allele resulting from gene conversion or recombination events between *GBA1* and its pseudogene *GBAP*. Often contains multiple pathogenic changes, leading to severe loss of GCase function and a severe GD phenotype.
**84insGG (frameshift)**	Severe (loss-of-function)	Essentially null or very low activity (frameshift/early truncation).	A severe loss-of-function variant that causes a frameshift mutation, leading to a premature stop codon and the production of a non-functional or severely truncated GCase enzyme, resulting in minimal to no activity.
***IVS2+1* G>A (splice)**	Loss-of-function/severe	Markedly reduced activity or aberrant splicing resulting in low/absent enzyme.	A splice-site mutation that disrupts the normal splicing of the *GBA1* mRNA. This leads to the production of abnormal mRNA transcripts and ultimately low or absent functional GCase enzyme, causing a severe loss-of-function phenotype.

GD: Gaucher Disease; PD: Parkinsons’ disease.

**Table 4:** *GBA1* increased GCase activity in PD-related animal and human cells models.

Study/ Model	Species/ Model	Manipulation/ Intervention	Outcome Measures	Main Outcomes	
**Migdalska-Richards [48,49]/L444P Gba1 knock-in mice**	Mouse (*L444P* knock-in; aged 24 months; and PFF injection studies)	*GBA1 L444P* mutation; sometimes combined with α-synuclein PFF	Increased total and insoluble α-synuclein, accelerated formation/spread of inclusions, motor impairments; reduced GCase activity.	Cortex p-α-synulein deposits: AT 45,000 ± 7000 vs. L444P/+ 96,000 ± 19,000 LB-like inclusions/mm^3^; ~30% decrease in GCase activity reported in brains of *L444P*/+ mice.	
**Rocha et al. [20] CBE (conduritol B epoxide) systemic GCase inhibition**	Mouse (systemic CBE administration)	Pharmacological inhibition of GCase using CBE	Reduced GCase activity; accumulation of GlcCer/GlcSph; increased α-synuclein, neuronal disturbances in vulnerable regions, motor deficits.	CBE 100 mg/kg x28 days: inhibited brain GCase activity (near-complete block in forebrain at 24 h post-final dose); GlcCer/GlcSph accumulated (measured by mass spectrometry); induced insoluble α-synuclein aggregates in SN; neuroinflammation and upregulation of complement C1q.	
**Viel et al. [50]/Venglustat preclinical**	Mouse models of *GBA*-related synucleinopathy (heterozygous *GBA* models)	Oral GCS inhibition with Venglustat (brain-penetrant)	Reduced GlcCer/GlcSph in brain and CSF; slowed α-synuclein accumulation in hippocampus; improved cognitive deficits in some models.	*GZ667161* (tool compound) in *GBA D409V/WT*: after 9 months plasma GlcCer reduced to 8 ± 6% of control; brain GlcCer to 58 ± 2% of control (*p* < 0.0001). Venglustat in *GBA D409V/D409V:* brain GlcCer 73 ± 2% remaining and plasma GlcCer 13 ± 1% remaining; brain GlcSph 63 ± 2% remaining; reduction in hippocampal aggregates (ubiquitin aggregates *p* = 0.03) and corrected novel object recognition performance.	
**Okai et al. [51]/AAV-mediated GBA1 gene therapy**	Mouse synucleinopathy models (AAV-PHP.B and other vectors)	AAV delivery of human *GBA1* to CNS	Restored GCase activity, reduced GlcSph/GlcCer, robust reduction in α-synuclein aggregates, rescue of motor/neurological phenotypes in preclinical studies.	AAV-*GBA1* overexpression: in vitro dissolution of p-α-synuclein aggregates; in vivo *A53T* M83 model: CBE increased HMW α-synuclein ~4-fold in striatum, AAV5-*GBA1* prior to CBE greatly reduced HMW α-synuclein accumulation; in GD mouse models, *GBA1* overexpression reduced GlcSph and rescued motor dysfunction (specific percent reductions vary by assay and region).	
**Keatinge et al. [52]/GBA1 knockout/mutant zebrafish**	Zebrafish *GBA1* c.1276_1298del (*GBA1*-/-)	Genetic knockout of *GBA1*	Accumulation of sphingolipids, neurodegeneration, motor deficits, reduced survival; recapitulates key GD and lysosomal phenotypes.	*GBA1* zebrafish: >30% reduction in dopaminergic neuronal counts at 12 weeks; accumulation of ubiquitin-positive inclusions; reduced survival (specific survival curves in main text).	
**Henderson et al. [53] GCase activity modulation and neuronal susceptibility**	Cellular models and in vivo mouse studies	Genetic or pharmacologic manipulation of GCase activity	Dose-dependent relationship between reduced GCase activity and increased pathogenic α-synuclein forms; altered neuronal susceptibility.	Dose-dependent relationship: studies show graded increases in pathogenic α-synuclein forms with decreasing GCase activity in cellular and mouse models (exact fold-changes vary by model; see Navarro-Romero et al. [14] and Grigor’eva et al. [54] for quantitative values including *N370S* 7.66% and *L444P* 15.85% residual activity in iPSC-derived neurons).	
**Silveira et al. [55]; Kopytova et al. [56]/Ambroxol in iPSC-derived neurons and animal models**	Human iPSC-derived dopaminergic neurons; rodent models	Ambroxol treatment (pharmacological chaperone)	Increased GCase activity, reduced GlcCer and α-synuclein levels, improved lysosomal markers and cell viability; some behavioural benefits in vivo.	Ambroxol treatment in patient-derived PBMC macrophages: increased GCase activity by 3.3-fold (GD) and 3.5-fold (*GBA*-PD) after 4 days (*p* < 0.0001); reduced HexSph by 2.1-fold (GD) and 1.6-fold (*GBA*-PD).	

AAV5: Adeno-associated virus serotype 5; AT: autopsied tissue; CBE: Carboxyamidotriazol; CNS: Central Nervous System; CSF: cerebrospinal fluid; GBA1: Glucosylceramidase beta 1; GD: Gausher disease; GCase: Glucocerebrosidase; GlcCer: glucosylceramide; GCS: Glucosylceramide synthase; GlcSph: glucosylsphingosine; HexSph: hexosylsphingosine; HMW: High molecular weight; iPSC: Induced pluripotent stem cell; LB: Lewy bodies; PD: Parkinsons’ disease; PFF: pre-formed fibrils, SN: substantia nigra.

## Data Availability

No new data were created or analyzed in this study. Data sharing is not applicable to this article.
